# Post-Chikungunya Rheumatoid Arthritis, Saint Martin

**DOI:** 10.3201/eid2103.141397

**Published:** 2015-03

**Authors:** Maud Foissac, Emilie Javelle, Simon Ray, Bruno Guérin, Fabrice Simon

**Affiliations:** Hospital Jacques Puel, Rodez, France (M. Foissac, S. Ray, B. Guérin);; Laveran Military Teaching Hospital, Marseille, France (E. Javelle, F. Simon)

**Keywords:** chikungunya, chikungunya virus, viruses, rheumatoid arthritis, post-chikungunya rheumatoid arthritis, chronic disease, Caribbean region, Saint Martin

**To the Editor:** In October 2013, autochthonous transmission of chikungunya was detected in the Caribbean area, which resulted in the current epidemic of chikungunya in the Western Hemisphere (*1*). The chikungunya virus strain that caused this epidemic belongs to the Asian lineage, not to the strain descending from the East/Central/South African (ECSA) lineage that spread in the Indian Ocean region after 2004. This ECSA lineage was reported mainly to cause long-lasting musculoskeletal and rheumatic disorders in chikungunya virus–infected patients (*2**–**8*). In 1984 in South Africa, Brighton and Simson reported post-chikungunya destructive polyarthritis (*6*). Twenty years later, the arthritogenic pathogenesis of viruses in the ECSA chikungunya virus lineage was confirmed after outbreaks in the Indian Ocean region (*2**–**5**,**7**,**8*).

Because >870,000 suspected cases of chikungunya have occurred during the past 12 months in the Western Hemisphere (http://www.paho.org/hq/index.php?option=com_content&view=article&id=9436), it is crucial to know whether infection with the epidemic Asian strain will cause chronic inflammatory and potentially destructive rheumatism. We report post-chikungunya rheumatoid arthritis from Saint Martin, the epicenter of the current epidemic.

A 70-year-old woman (artist–painter) in Saint Martin sought treatment in June 2014 for joint pains and disabilities persisting after chikungunya. Her medical history included high blood pressure, hypothyroidism, and 3 dengue infections. During October 2013, the patient had high-grade fever, intense fatigue, and a maculopapular troncular exanthema without lymphadenopathy. Five days later, she had distal polyarthritis (joint pain and swelling) in interphalangeal joints, wrists, and ankles without plantar involvement. Recent infection with chikungunya virus was confirmed (IgM and IgG against chikungunya virus was detected in 2 blood samples), and recent dengue was excluded according to the criteria of the National Reference Center on Arboviral Diseases (http://www.niaid.nih.gov/labsandresources/resources/dmid/wrceva/Pages/default.aspx).

Despite initial brief improvement, the patient never totally recovered and subsequently chronic polyarthritis developed, which involved >10 joints, including interphalangeal joints, wrists, and knees. Nonsteroidal antiinflammatory drugs did not relieve the diffuse pain, stiffness, and swelling. She was given oral corticotherapy (20 mg/day) beginning in January 2014. She was referred to another hospital in France 5 months later because of treatment failure. She reported continuous pain in the left knee and wrists and multiple tenosynovitis on flexors and extensors of the fingers (Figure). She did not report any fever or axial, shoulder, or hip pain. Radiographs of the involved joints showed no abnormalities.

**Figure:**
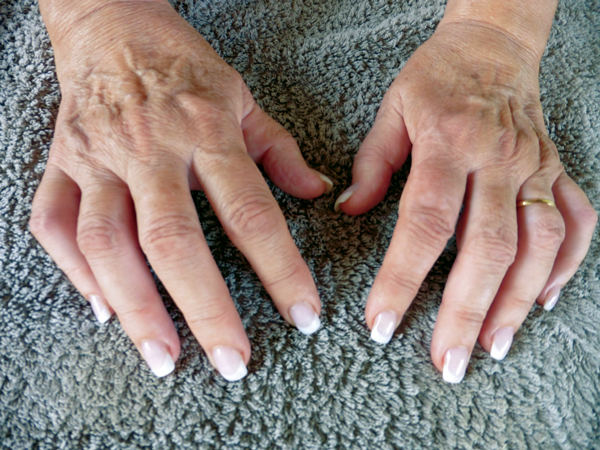
Swollen and stiff hands of a 70-year-old woman with post-chikungunya rheumatoid arthritis 10 months after acute infection with chikungunya virus, Saint Martin.

The patient had mild inflammation (C-reactive protein level 13 mg/L, fibrinogen level 3.4 g/L) but no specific autoimmunity (results were negative for anticitrullinated peptide antibodies, rheumatoid factor, antineutrophil cytoplasmic antibodies, and antinuclear antibodies). Serologic results for viruses other than chikungunya virus were negative or indicated past vaccination. The patient’s condition met the 2010 American College of Rheumatology/European League against Rheumatism criteria for rheumatoid arthritis (https://www.rheumatology.org/practice/clinical/classification/ra/ra_2010.asp), and the only cause observed for this disease was acute chikungunya. For this corticosteroid-resistant, seronegative, and nondestructive post-chikungunya rheumatoid arthritis, methotrexate was prescribed at a weekly low dose after exclusion of contraindications, but the patient was not followed-up after she returned to Saint Martin.

The reported case was caused by chikungunya virus infection during an epidemic in Saint Martin in October 2013. This unfavorable post-chikungunya outcome of chronic inflammatory rheumatism 8 months later indicates a probable course of post-chikungunya disorders in the Western Hemisphere, as has already been observed in Africa and Asia. Previous outbreaks in Réunion and India offer insights regarding patients’ post-chikungunya chronic status with long-lasting pain and disability, impaired quality of life, and extensive treatment (*2**,**3**,**9*).

The spectrum of post-chikungunya rheumatic and musculoskeletal disorders includes multiple tendinitis and tenosynovitis, plantar fasciitis, mechanical disbalance in susceptible joints, tunnel syndromes, edematous polyarthralgia, rheumatoid arthritis, and psoriatic arthritis (*2**,**4**,**5*). Although the proportion of patients with chronic disease has decreased, post-chikungunya chronic inflammatory rheumatism, mostly rheumatoid arthritis, develops in ≈5% of these patients (*8*). These patients had a poor prognosis and were given disease-modifying anti-rheumatic drugs (DMARDs), despite the postinfectious origin of rheumatism (*4**,**5*). Patients with post-chikungunya rheumatoid arthritis should benefit from methotrexate, which is recommended for treatment of classic rheumatoid arthritis (*10*).

In our experience, resistance to or dependence on corticosteroids beyond the third month after disease onset is highly evocative of post-chikungunya chronic inflammatory rheumatism. This finding requires early treatment with DMARDs to control the inflammatory process, prevent bone erosions, and prevent inevitable side effects of prolonged corticotherapy. To date, the efficacy of different DMARDs for treatment of post-chikungunya chronic inflammatory rheumatism has not been evaluated. Therefore, physicians should follow the international guidelines for treatment of classic rheumatoid arthritis and psoriatic polyarthritis, which recommend methotrexate as first-line treatment for patients fulfilling chronic inflammatory rheumatism criteria after 3 months of evolution.

We found that the Asian strain of chikungunya virus has induced arthritic disorders in the Western Hemisphere. Thus, a possible increase in post-chikungunya rheumatoid arthritis should not be overlooked. Physicians and public health authorities should prepare a response to the patients’ post-chikungunya stage in the epidemic areas. Clinical vigilance is recommended to identify patients with unfavorable outcomes 3 months after disease onset and for those in whom post-chikungunya chronic inflammatory rheumatism develops and who require specific treatment. Detailed guidelines for diagnosis and treatment of these patients with chronic rheumatoid arthritis are needed.
